# Successful revascularization with percutaneous coronary intervention using a combination of the subintimal transcatheter withdrawal technique and coronary artery fenestration for spontaneous coronary artery dissection

**DOI:** 10.1002/ccr3.5045

**Published:** 2021-11-07

**Authors:** Shintaro Matsuura, Kanichi Otowa, Michiro Maruyama, Kazuo Usuda

**Affiliations:** ^1^ Department of Cardiology Toyama Prefectural Central Hospital Toyama Japan

**Keywords:** computed tomography angiography, coronary balloon angioplasty, intravascular ultrasonography, myocardial infarction, percutaneous coronary intervention

## Abstract

The combination of the STRAW technique and coronary artery fenestration using a cutting balloon could be effective in SCAD patients, especially with dissection to the distal end of the coronary artery.

## Graphical abstract

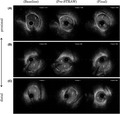

## 1 INTRODUCTION

We present a case of successful revascularization for spontaneous coronary artery dissection (SCAD) using coronary artery fenestration followed by the subintimal transcatheter withdrawal (STRAW) technique. The combination of the STRAW technique and coronary artery fenestration with cutting balloon angioplasty may be a new treatment option for SCAD patients requiring revascularization.

Spontaneous coronary artery dissection (SCAD) has emerged as an important cause of acute coronary syndrome, particularly in young woman.^1^, ^2^, ^3^ Although recent studies have demonstrated that conservative therapy is appropriate for the initial management of SCAD, revascularization should be considered in a patient with ongoing ischemia, high‐risk anatomy, or hemodynamic instability.^4^, ^5^ Percutaneous coronary intervention (PCI) for SCAD treatment is associated with an increased risk of complications and suboptimal outcomes; however, several case reports have shown the efficacy of coronary artery fenestration with cutting balloon angioplasty.^6^, ^7^, ^8^ Here, we present the case of a patient with SCAD whose ischemia was not corrected with coronary artery fenestration alone but was successfully revascularized by the additional use of the subintimal transcatheter withdrawal (STRAW) technique.^9^, ^10^

## 2 CASE PRESENTATION

**FIGURE 1:**
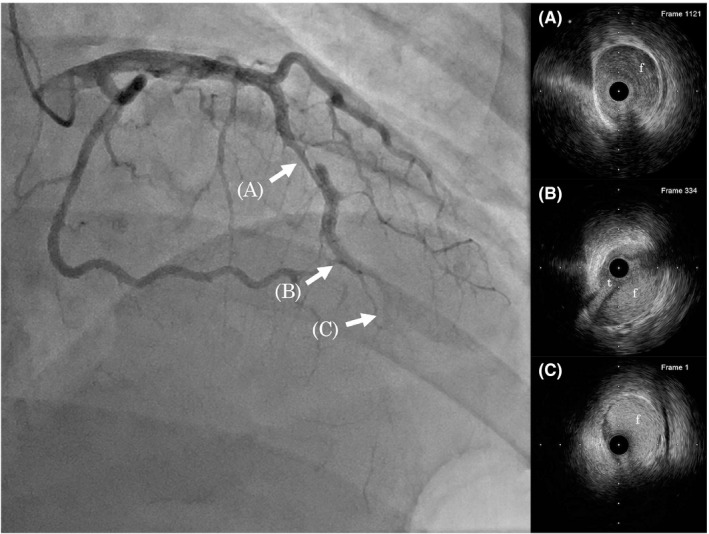
Baseline left coronary angiography and IVUS images. The angiography (left) shows subtotal occlusion in the middle part of the LAD, and there appeared to be a contrast delay distally. IVUS images (right) demonstrate the false lumen compressing the true lumen in the middle (A) to distal (B and C) LAD. t = true lumen, f = false lumen

**FIGURE 2:**
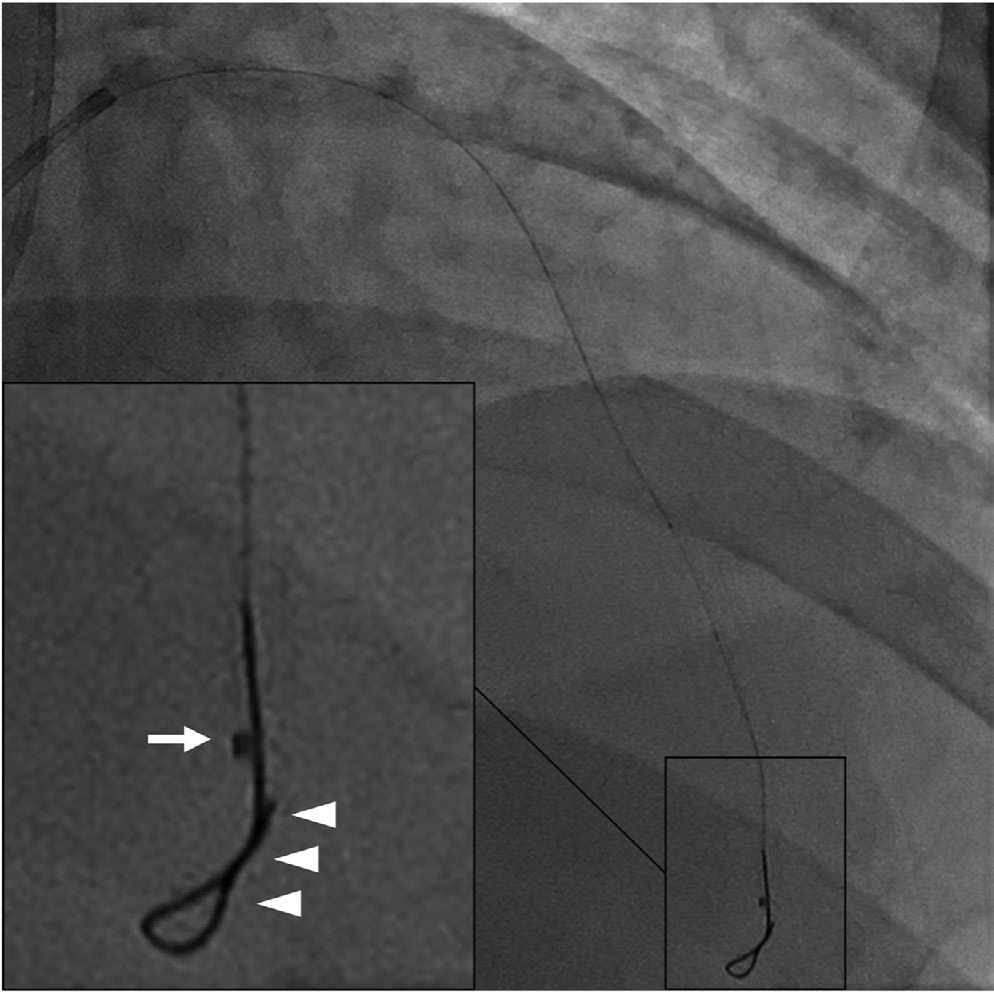
STRAW technique using a microcatheter. The first guidewire (arrowhead) was placed into the true lumen, and the microcatheter (arrow) advanced into the false lumen at the far distal part of the LAD. With the application of negative pressure using a syringe, the hematoma was aspirated from the tip of the microcatheter, while gradually moving the microcatheter position to the proximal part

**FIGURE 3:**
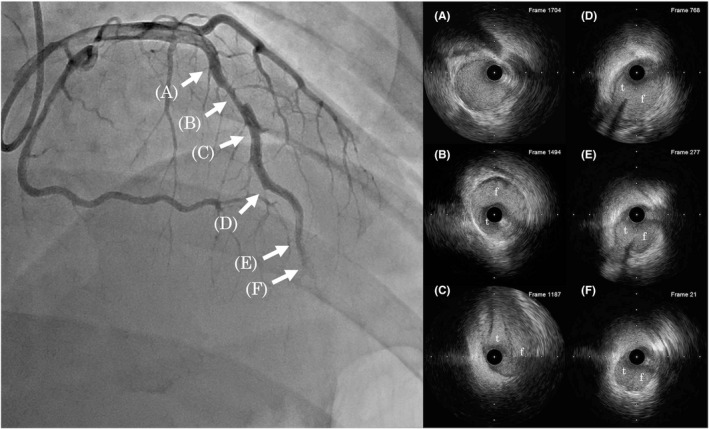
Final coronary angiography and IVUS images. The angiography (left) shows improvement in the coronary artery blood flow from TIMI 1–2. In addition, small side branches at distal LAD can be observed. IVUS images (right, A‐F) demonstrate multiple connections between the true lumen and the false lumen in the middle (D) to distal (E) part of the lesion, with true lumen enlargement (D‐F). t = true lumen, f = false lumen

**FIGURE 4:**
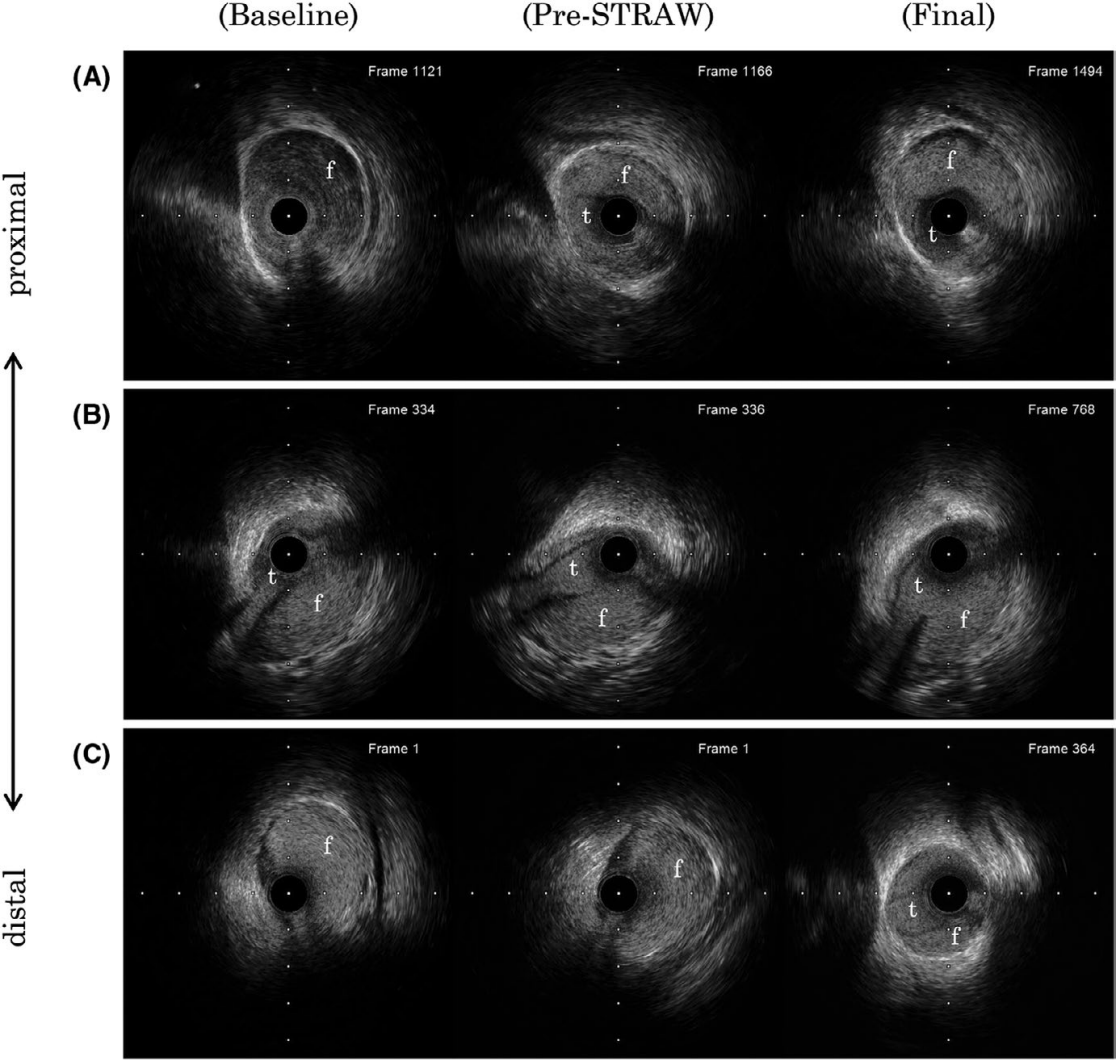
Comparison of the IVUS images during each procedure (baseline, pre‐STRAW, final). IVUS images recorded at approximately the same site are placed side by side. (A) Proximal, (B) middle, (C) distal part of the lesion. t = true lumen, f = false lumen

A 31‐year‐old woman with a history of smoking but no prior coronary artery disease was admitted to our hospital at 20 weeks of gestation for impending preterm labor. At 36 weeks of gestation, a cesarean section was performed. However, 2 days thereafter, she developed chest pain with anterior ST‐segment elevation on electrocardiogram, indicative of acute myocardial infarction. Emergency coronary angiography (CAG) showed subtotal occlusion in the middle part of the left anterior descending artery (LAD), and there appeared to be a contrast delay distally. In order to confirm the cause of subtotal occlusion, an IVUS examination was performed (Figure 1). A 6‐French guiding catheter (Hyperion SPB3.5, ASAHI INTECC CO, LTD) was engaged in the left coronary artery by the right transradial artery approach. The IVUS (Opti‐Cross HD, Boston Scientific, Inc) then demonstrated that the false lumen compressed the true lumen from the middle to distal part of the LAD. Based on the findings of the CAG and IVUS, she was diagnosed with type‐1 SCAD. The IVUS catheter was advanced as far distally as possible; however, the distal end of the false lumen could not be identified. Due to ongoing chest pain and persistent ST elevation on the electrocardiogram, we decided to perform primary PCI. First, we attempted coronary artery fenestration using cutting balloon angioplasty. A 3.5‐mm cutting balloon (Wolverine, Boston Scientific, Inc) was advanced into the distal LAD where the IVUS catheter could be advanced and then dilated. The IVUS findings after cutting balloon angioplasty showed the creation of some reentries; however, the true lumen at the distal LAD was still compressed by the hematoma in the false lumen. In order to make an additional fenestration at a more distal site, we attempted to advance a 2.5‐mm cutting balloon (Wolverine, Boston Scientific, Inc); however, this attempt was unsuccessful. Therefore, we then applied the STRAW technique. A second guidewire (SUOH03, ASAHI INTECC CO., LTD) was intentionally advanced into the false lumen under real‐time IVUS guidance, and a microcatheter (FINECROSS GT, TERUMO Corp) was advanced into the false lumen at the far distal part of the LAD. After the guidewire was removed, we aspirated blood from the tip of the microcatheter while gradually moving the microcatheter position to the proximal side; then, ST‐segment elevation improved, and chest pain disappeared (Figure 2). We attempted to advance a 2.5‐mm cutting balloon again, and it was able to advance more distally than before the STRAW technique; thereafter, coronary artery fenestration was performed at the distal LAD. The IVUS findings after the STRAW technique and the additional coronary artery fenestration at the distal LAD showed an enlarged true lumen and multiple reentry formations. Although there were several stenoses in the middle part of the LAD due to compression by the false lumen, coronary artery blood flow improved to Thrombolysis in Myocardial Infarction (TIMI) flow grade 2 (Figures 3 and 4). Considering the absence of ongoing ischemia and the patient's age and gender, we decided to complete the procedure without stenting.

**FIGURE 5:**
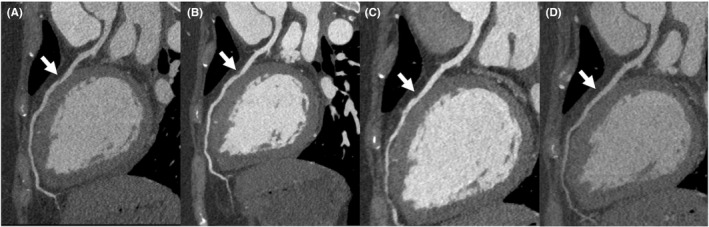
Coronary computed tomographic angiography images for follow‐up after PCI. Each image shows multiplanar reconstructed images of LAD, (A) 1 week, (B) 2 weeks, (C) 2 months, and (D) 10 months after PCI. The flap and coronary artery stenosis in the middle of the LAD (arrow) gradually disappeared

Since a small acute pulmonary embolism was incidentally found on CT before PCI, the antithrombotic drugs, clopidogrel (75 mg once daily) and apixaban (10 mg twice daily for 1 week, followed 5 mg twice daily), were started after the PCI procedure. One month later, apixaban was discontinued after confirming improvement of the pulmonary embolism and antiplatelet monotherapy was used thereafter. The post‐procedural course was good and uneventful until 10 months thereafter. Due to the risks of coronary artery dissection with invasive coronary angiography, we followed up the patient with coronary computed tomographic angiography; the flap and the stenosis at the middle part of the LAD disappeared after 10 months of PCI (Figure 5).

## 3 DISCUSSION

Previous observational studies have consistently shown that PCI for SCAD treatment is associated with an increased risk of complications and suboptimal outcomes.^1^, ^2^, ^3^, ^4^, ^5^ However, owing to ongoing ST‐segment elevation, we performed primary PCI for this patient.

Some case reports have shown the efficacy of coronary artery fenestration with cutting balloon angioplasty to decompress the hematoma and avoiding longitudinal extension.^6^, ^7^, ^8^ We also attempted this technique; however, our attempt was unsuccessful. We believe the following reasons were responsible for the failure of this attempt: 1) the cutting balloon could not advance sufficiently distally because the true lumen was severely compressed by the false lumen and 2) the dissection had reached the distal end of the LAD. Therefore, coronary artery fenestration could not be made at the distal end of the false lumen, and the compression of the true lumen was not released by the remaining hematoma in the false lumen. In order to overcome these problems, we used the STRAW technique.^9^, ^10^

The STRAW technique was reported as an option to decompress the distal true lumen and enable successful reentry for antegrade in chronic total occlusion cases. In the original method, an over‐the‐wire balloon was positioned in the proximal vessel at the site of origin of the dissection plane. Following balloon inflation, a 10‐ml syringe was attached to the proximal lumen port on the negative pressure.^10^ In addition, the case report of iatrogenic coronary artery dissection from our hospital has shown that aspiration using a microcatheter could reduce the hematoma in the false lumen.^11^ In that report, the guidewire could not pass into the true lumen because it was compressed by the hematoma in the false lumen due to catheter‐induced coronary artery dissection. Thus, we succeeded in passing the guidewire into the true lumen by aspirating the hematoma in the false lumen using a microcatheter and releasing the pressure in the true lumen. In this case, we performed microcatheter aspiration to successfully improve the ongoing ischemia. Furthermore, it became possible to deliver a cutting balloon to a more distal part of the LAD, and we succeeded in performing the coronary artery fenestration at a sufficiently distal side. This avoided re‐expansion of the hematoma in the false lumen and re‐compression of the true lumen at the distal LAD. We believe that aspiration using a microcatheter offers the advantage of safe advancement of the aspiration device to the distal part of the false lumen. However, if microcatheter aspiration is insufficient, it may be helpful to perform aspiration during balloon occlusion in the proximal vessel, referring to the original method.

Several PCI techniques for SCAD with or without stenting have been advocated; however, there are limited comparative outcome data. Importantly, PCI in SCAD should focus on restoring the TIMI flow grade and stabilizing the patient clinically rather than restoring normal coronary artery structure.^2^ In this case, we decided to perform stent‐less PCI because ST‐segment elevation and TIMI flow grade improved, and the patient was a young woman. Observational data have shown that spontaneous healing occurs in most SCAD patients,^3^ and in the present case, coronary computed tomographic angiography still showed healing of the SCAD lesion.

This new PCI strategy that combined the STRAW technique and coronary artery fenestration using a cutting balloon appeared effective in SCAD patients; however, a more large‐scale study is required to confirm the safety and efficacy of this method.

## 4 CONCLUSION

A combination of the STRAW technique and coronary artery fenestration with cutting balloon angioplasty could be effective in SCAD patients, with dissection to the distal end of the coronary artery. However, a more large‐scale evaluation is required to confirm the safety and efficacy of this method.

## CONFLICT OF INTEREST

The authors declare that there is no conflict of interest.

## AUTHOR CONTRIBUTIONS

SM: involved in management of the patient and wrote the article. KO, MM, and KU: involved in critically reviewing and revising the manuscript. All authors: approved the final version of the case report or submission to *Clinical Case Reports*.

## ETHICAL APPROVAL

The study was published with written consent of the patient.

## CONSENT

Published with written consent of the patient.

## Data Availability

The data that support the findings of this study are available from the corresponding author upon reasonable request.
